# Assessing a “Least-Concern” Red List Tree Species from Madagascar Used in Traditional Medicine: *Morella spathulata* (Myricaceae) Phyto-Compounds and Anti-Inflammatory Properties

**DOI:** 10.3390/plants13202899

**Published:** 2024-10-17

**Authors:** Annachiara Fioccardi, Dario Donno, Zoarilala Rinah Razafindrakoto, Nantenaina Tombozara, Sylvia Henintsoa, Elyna Mahitasoa, Valeria Torti, Marcellin Solofoniaina, Lorenzo Rosso, Giovanni Gamba, Charles Andrianjara, David Ramanitrahasimbola, Gabriele Loris Beccaro

**Affiliations:** 1 Dipartimento di Scienze Agrarie, Forestali e Alimentari, Università degli Studi di Torino, 10095 Grugliasco, TO, Italy; dario.donno@unito.it (D.D.); hnsylvia98@gmail.com (S.H.); lorenzo.rosso@unito.it (L.R.); giovanni.gamba@unito.it (G.G.); gabriele.beccaro@unito.it (G.L.B.); 2 Institut Malgache de Recherches Appliquées, B.P. 3833, Antananarivo 101, Madagascar; zo_ari_lala@yahoo.fr (Z.R.R.); nzara89@gmail.com (N.T.); elynamahitasoa@gmail.com (E.M.); yvesmickaelsolofoniaina@gmail.com (M.S.); charles.andrianjara@gmail.com (C.A.); ramanitrahasimboladavid@gmail.com (D.R.); 3 Dipartimento Scienze della Vita e Biologia dei Sistemi, Università degli Studi di Torino, 10123 Torino, TO, Italy; valeria.torti@unito.it

**Keywords:** *Morella spathulata*, antioxidant, traditional medicine, analgesic activity, anti-inflammatory effects, phytochemicals

## Abstract

*Morella spathulata* (Myricaceae family) is a common plant from Madagascar and is present on the IUCN Red List of threatened species classified at the ’least concern’ level, used by the local population to treat numerous illnesses and pain. Despite its frequent use, comprehensive phytochemical and pharmacological research on the species is limited. This study evaluated the antioxidant, analgesic, and anti-inflammatory properties, as well as the toxicity of methanol extracts from the leaves (MS_L) and bark (MS_B) of *M. spathulata*. The research involved the analysis of nutritional traits such as sugars, organic acids, vitamin C, polyphenolic content (TPC) and the main phytochemicals by HPLC analysis. Antioxidant capacity was assessed through DPPH and FRAP assays. Analgesic and anti-inflammatory activities were evaluated using acetic acid-induced writhing and carrageenan-induced paw oedema tests in mice. The results showed a high content of phenolic and bioactive components in the leaf and bark extracts, associated with antioxidant, analgesic and anti-inflammatory properties. The interaction of key compounds such as ferulic acid and ellagic acid with proteins involved in pH regulation and immune modulation provides clues to the mechanisms underlying the therapeutic effects. However, conservation efforts are crucial due to habitat loss and illegal logging, and further studies are needed to fully explore the plant’s therapeutic potential.

## 1. Introduction

The application of medicinal plants in the traditional African healthcare system is one of the oldest and most diverse therapeutic approaches. Throughout history, traditional medicine has consistently served as an alternative solution for various illnesses [1]. More than 90% of the world’s population uses traditional medicines based on natural products as primary health care. Among the actual pharmaceutical products and drugs, almost 73% are derived from natural products, including active substances extracted from plants [2]. Especially in developing countries, such as Madagascar, where access to modern medical resources is often limited or financially expensive, the use of health-promoting remedies derived from natural resources is very important [3]. In addition, the deep-rooted cultural familiarity, traditionalism, and perceived safety of traditional medicine are influential, making it the preferred choice among the local population [4,5].

Madagascar has an interesting natural heritage of medicinal plants, which contribute to its rich pharmacopoeia, thanks to its wide range of plant species. The highly abundant species is a result of Madagascar’s detachment from Africa approximately 170 million years ago, and then by the Indian subcontinent nearly 88 million years ago, making it the largest reserve of endemism and biodiversity on the planet [4,6]. This botanical diversity is a valuable resource for traditional and modern medicine. In Malagasy traditional medicine, several plants are used to cure gastrointestinal disorders such as diarrhea and intestinal parasites [4]. Furthermore, this conventional practice is also applied to the prevention of respiratory infections like bronchitis and colds, which are common in rural areas of the country [7]. This traditional practice becomes increasingly important in the context of rising drug costs and resistance to diseases, including malaria, bacterial infections, and sexually transmitted diseases. As a result, there is a new focus on exploring traditional medicine as a promising strategy for the discovery of new chemical compounds against these health challenges [8]. However, the spontaneous collection of medicinal plants in forests causes a negative impact on the local ecosystem, resulting in a significant reduction in biodiversity [9]. Nonetheless, the controlled propagation of these plant species presents a promising solution to replace the random collection of plants [10]. Propagation and dedicated cultivation can reduce overharvesting and ensure high-quality material production, emphasizing the importance of characterizing the Malagasy plant biodiversity by phytochemical and pharmacological analysis.

*Morella spathulata* (Mirb.) Verdc. and Polhill (Myricaceae family), also known as *Myrica spathulata* Mirb, is widespread across eastern Madagascar, from north to south provinces [11]. It is also present in Mozambique (in the Sofala region, approximately 90 km north of Beira) and Tanzania (Pemba), up to 1000 m above sea level [12], even if the botanical classification in this region is still unclear. *M. spathulata* is a shrub to small tree up to 12 m high. It occurs in grasslands with marginal or high-water tables on peaty soils, riverbanks, and sandy coast plains, frequently associated with *Erica* spp. [13,14]. The leaves are oblanceolate to strictly oblong-elliptical, closely obovate or obovate-spatulate, 2.5 to 9 cm long and 1.5 to 3.5 cm wide, rounded or sometimes notched at the apex, attenuate-decurrent at the base, leathery, smooth, and often shiny above, with small thick, consistent red-brown scales below, with 10 to 20 lateral veins on all side, rather inconspicuous below. The inflorescences are glandular, oval, and obtuse, varying in length from 1.5 to 3 cm. The fruits are ovoid and globose, 4 to 5 mm in diameter [15]. This species represents an example of the exploitation of natural resources and loss of biodiversity. Indeed, although *M. spathulata* is distributed and present in protected areas, it is threatened by illegal logging, agriculture, and the slash-and-burn practice. Currently listed as ‘least concern’, its importance in traditional medicine is significant [16]. The leaves and barks of this species are prepared as infusion or decoction for various medicinal purposes on the east coast of Madagascar, including a mouthwash to relieve toothache, pain, and inflammation occurring during the sickle cell patient crisis [12]; the leaves were chewed and applied to wounds. Despite its medicinal virtues, this species has not yet been subjected to comprehensive phytochemical or pharmacological investigations. Therefore, the objective of this survey is to examine the antioxidant, analgesic, and anti-inflammatory activities, and acute toxicity, along with the phytochemical and nutritional traits of *M. spathulata* aerial parts extracts.

## 2. Results and Discussion

### 2.1. Total Polyphenolic Contents (TPC)

Plant phenols are a notable category of compounds known for their role as primary antioxidants or free radical terminators. Indeed, assessing their cumulative amount is a valuable tool for discerning plants and extracts with potential commercial prospects [17]. Furthermore, in several studies, as well as in the present work, the spectrophotometric method for TPC evaluation has been used in association with chromatographic analysis to strengthen and validate the results, establishing its credibility as a complementary technique [18,19]. In this study, TPC for *Morella spathulata* extracts ranged from 8913.58 ± 1940.88 mg GAE/100 g DW in leaves to 10,177.81 ± 1241.09 mg GAE/100 g DW in bark. These values are very high if compared to other species, such as *Morella parvifolia* (Benth.) Parra-Os., native to South America; in a study conducted by Puertas-Mejía, M. A. et al. [20], they identified a TPC value of 51.41 ± 0.44 mg GAE/100 g DW in the leaves of *M. parvifolia*. The bark also showed a high value of TPC (10,177.81 ± 1241.09 mg GAE/100 g DW), which may be related to the high accumulation of polyphenolic components in bark tissues during the lignification processes, as confirmed by many studies [21,22].

### 2.2. Phytochemical Fingerprint

**Figure 1:**
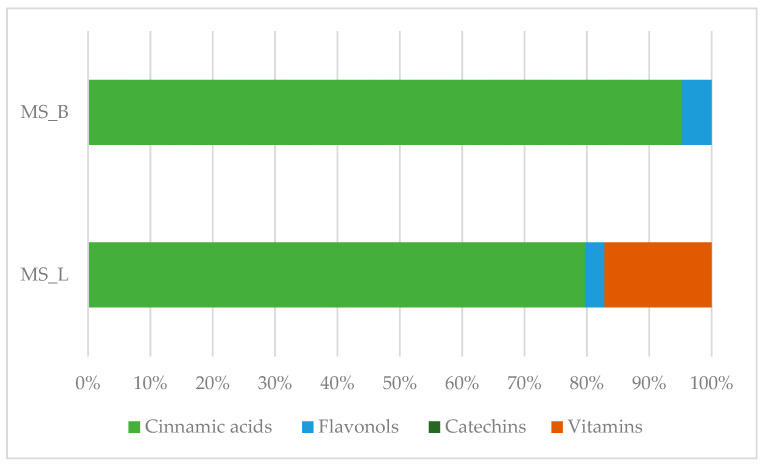
Percentage contribution of phenolic compounds and vitamins in the analyzed samples. MS_L: *M. spathulata* leaf extract; MS_B: *M. spathulata* bark extract. Mean values are shown (N = 3).

In this paper, 24 bioactive components and nutrients were chosen for their relevance in human health [23]. These compounds were used as markers to determine the HPLC fingerprint (Table 1). In general, the number of identified bioactive compounds was higher in the leaves than in the bark (eight vs. seven). Unlike spectrophotometric techniques, liquid chromatography offers the possibility of quantifying specific polyphenolic targets. Several markers, known for their high biological activity, were selected, and their higher presence in leaves could be due to the leaf structural differences compared to bark, which may present a lower concentration of these compounds. The value of bioactive substances with high antioxidant capacity in leaves and bark was examined by grouping and comparing the amount of the selected phytochemicals: polyphenolic components (flavonols, benzoic acids, cinnamic acids, and catechins) and vitamin C (dehydroascorbic and ascorbic acids) (Figure 1). Regarding cinnamic acids, leaf extract was the most significant contributor, with a mean value of 67.16%, as opposed to the bark, with a value of 49.67%. Flavonols, in leaves and bark extract contributed to the antioxidant compounds almost equally (with values of 2.63% and 2.50%, respectively). Benzoic acids were most abundant in bark extract (22.11%) rather than in leaves (19.05%). Vitamin C showed the highest value in leaf extract with a value of 14.49%.

More in detail, within the class of cinnamic acids, the only compound quantified was ferulic acid, with a value of 67.16 ± 0.73 mg/100 g DW in the leaves and 49.67 ± 2.09 mg/100 g DW in the bark. Ferulic acid is known for its antimicrobial and antioxidant properties [24,25]. These properties may support its potential use in traditional medicine, especially considering the important quantities found. In the class of flavonols, only isoquercitrin was quantified in the bark (2.5 ± 0.46 mg/100 g DW) and only rutin was detected in the leaves with a value of 2.63 ± 1.66 mg/100 g DW. Among the class of benzoic acids, only ellagic acid was quantified with a value of 19.08 ± 1.5 mg/100 g DW in the leaf and 22.11 ± 2.26 mg/100 g DW in the bark. Plants produce ellagic acid by the hydrolysis of tannins [26]. Tannins concentration is generally greater in the bark compared to other parts of the plant [27], which likely accounts for the higher levels of ellagic acid that have been quantified in the bark. The ellagic acid content found in the leaves was similar to other methanolic extracts of endemic Malagasy plants (e.g., *Tina striata* with a value of 19.33 ± 2.96 mg/100 g DW [28], and *Phyllarthron bojeranum* with a value of 18.16 ± 6.5 mg/100 g DW [29]. The same is also true for the bark value (22.11 ± 2.26 mg/100 g DW), which can be compared to that reported in *Schefflera bojeri* (24.46 ± 10.54 mg/100 g DW) [30]. Ellagic acid plays a key role in anti-atherogenic, anti-inflammatory, and neuroprotective actions. In addition to its function as a liver and skin protector and as an anti-cancer agent, it influences cellular processes and DNA damage protection and has anti-inflammatory properties [31]. Furthermore, a positive correlation (R = 0.998; *p* < 0.05) was noted between ellagic and ferulic acids, suggesting a potential interaction between these two compounds in *M. spathulata*. This correlation indicates the complex biochemical relationships throughout the plant, which might affect their synergistic effects or shared metabolic pathways.

To further investigate the correlation found between ellagic acid and ferulic acid, the identification of their molecular targets and pharmacokinetic profiles was carried out using the Swiss Target Prediction platform (http://www.swisstargetprediction.ch/, accessed on 8 April 2024), as previously reported in Fawzi M. et al. [32]. Based on the platform, it was predicted that the targets of ferulic acid are carbonic anhydrases (CA2), a key enzyme for acid–base balance and carbon dioxide transport [33]. CA2, present in tissues such as the liver, also plays a crucial role in pH regulation [34]. The activity of CA2 has an impact on conditions related to acid–base imbalance, and its interaction with ferulic acid may suggest potential anti-inflammatory effects [35]. In comparison, ellagic acid was found linked to the G protein-coupled receptor 35 (GPR35). It is present in various organs, among them the digestive tract and immune cells [36], and the interaction of ellagic acid with GPR35 may influence immune regulation and inflammation, contributing to anti-inflammatory and neuroprotective properties. Moreover, the role of GPR35 in gastrointestinal health also suggests a potential impact on digestive functions [37,38]. Catechins have not been detected. Many studies confirmed that catechins are highly unstable in sunlight conditions [39,40]. In this case, plant materials of *M. spathulata* were dried in sunlight according to local traditions, which may have influenced the degradation of catechins. About vitamin C, ascorbic and dehydroascorbic acids were evaluated. In this study on *Morella spathulata*, only ascorbic acid was found in the leaves with a value of 14.49 ± 0.19 mg/100 g DW. Vitamin C content varies depending on species and environmental conditions and among different meristematic tissues and organs, generally being high in flowers, leaves, and young fruits and low in non-photosynthetic tissues such as stems, roots, and bark [41,42]. For this reason, vitamin C may have been only identified in the leaves and not in the bark. Vitamin C functions as an antioxidant safeguarding the body from oxidative stress and preserving the immune system [43]. Additionally, it has the capability of neutralizing radicals and damaged lipids and DNA in the blood, which can lead to disease [44].

Organic acids and sugars are reported in Table 2. Both in plant materials of *M. spathulata*, quinic acid was identified; a value of 48.57 ± 0.68 g/100 g DW was detected in the leaves and 21.50 ± 0.75 g/100 g DW in the bark. In vitro and in vivo pharmacological studies have demonstrated that quinic acid possesses several biological activities, including antioxidant, antitumor, antimicrobial, antiviral, and analgesic properties [45]. The antioxidant activity of this molecule was also confirmed by the correlation (R = 0.999; *p* < 0.05) with antioxidant capacity (AOC). In addition, it stimulates insulin secretion by releasing intracellular Ca^2+^ and increasing the NAD(P)H/NAD(P)+ ratio, contributing to anti-diabetic effects [46]. Instead, citric acid was identified in the bark extract with a value of 3.55 ± 0.46 g/100 g DW. Citric acid is an excellent harmless disinfectant against various viruses [47]. Its presence may validate the use of *M. spathulata* in traditional medicine as a wound disinfectant. In addition, citric acid was indicated as a potent antioxidant [48], confirmed by the positive correlation (R = 0.997) with the values of free radical scavenging activity (DPPH).

*M. spathulata* showed the highest sugar content in the leaves and bark for glucose (24.81 ± 0.76 g/100 g DW and 31.44 ± 1.22 g/100 g DW, respectively). Compared to other Malagasy plants studied, which had glucose values below 1 g/100 g DW [28], a significantly higher glucose content was observed here. The high glucose content quantified in this plant could be beneficial for diabetics in hypoglycemic situations, representing a source of sugar to consume when necessary. Furthermore, according to the Swiss Target Prediction platform (http://www.swisstargetprediction.ch/, accessed on 8 April 2024), the glucose molecule was identified to target the protein Cholinergic Receptor Nicotinic Alpha 7 Subunit (CHRNA7), a receptor crucial for the anti-inflammatory cholinergic pathway associated with autoimmune disorders [49]. It also contributes to cell proliferation and angiogenesis in lung and pancreatic cancers and serves as a key component of the cholinergic nervous system in the brain [50,51]. Fructose was detected in similar levels both in leaf and bark extracts, with values of 1.26 ± 0.04 g/100 g DW and 1.75 ± 0.03 g/100 g DW, respectively. It is interesting to note that fructose can act as an antioxidant due to its reducing capacity [52]. Sucrose was only detected in leaf extract with a value of 6.04 ± 0.54 g/100 g DW, as it is one of the main products of photosynthesis in leaves [53]. In general, contrary to other Malagasy flora, which are generally characterized by a lower carbohydrate content, *M. spathulata* could be a promising food resource, especially in countries with high malnutrition rates, due to its considerable sugar levels.

### 2.3. In Vitro Antioxidant Capacity (DPPH and FRAP)

In *M. spathulata* bark and leaves, the antioxidant properties were evaluated by two different tests: the free radical scavenging activity test (DPPH) and the ferric reducing antioxidant power test (FRAP). All the results of the antioxidant capacity are shown in Table 3. The DPPH is a rapid and simple method to test antioxidant activity based on the ability of some molecules to perform their capacity of being radical scavengers and hydrogen donors. In this case, the scavenging potential of extracts is based on the DPPH solution discoloration from purple (free radical stable in methanol) to yellow (DPPH reduced by radical scavenger) [54].

As shown in Table 3, maceration yielded 9.84% of MS_B and 3.90% of MS_L. The IC_50_ values were 26.78 ± 0.42 mg/mL for MS_L and 29.74 ± 0.16 mg/mL for MS_B. These values were close to those of gallic acid (IC_50_ = 21.78 ± 0.11 mg/mL), which represented the positive control used. MS_L and MS_B were more active compared to other Malagasy medicinal plants as reported in previous studies. For example, the aerial parts of *Vaccinium secundiflorum* were characterized by an IC_50_ value of 76.07 ± 1.08 mg/mL [55], while the extracts of *Uapaca bojeri* showed lower IC_50_ values (47.36 ± 3.00 and 33.32 ± 0.69 mg/mL, respectively, for leaves and bark) [54]. The *Lygodium lanceolathum* and *Imperata cylindrica* aerial parts presented IC_50_ of 107.05 ± 3.41 mg/mL and IC_50_ of 192.07 ± 0.78 µg/mL, respectively [56,57]. The DPPH values on *M. spathulata* antioxidant capacity were also confirmed by FRAP results. The FRAP values were 227.89 ± 12.53 mmol Fe^2+^/kg of DW for the leaves and 376.18 ± 115.69 mmol Fe^2+^/kg of DW for the bark. Such values are higher than those found in another Malagasy medicinal plant, *Uapaca bojeri*, where the leaves and bark had values of 69.20 ± 1.41 mmol Fe^2+^/kg of DW and 70.17 ± 9.53 mmol Fe^2+^/kg of DW, respectively [54]. These results confirmed the importance of *M. spathulata* in popular tradition as a source of potentially beneficial treatments and underlined the necessity of conserving this plant resource.

### 2.4. Anti-Inflammatory and Analgesic Activity

#### 2.4.1. Carrageenan-Induced Paw Oedema

Reducing oedema rates exerted by MS_L and MS_B are reported in Table 4. The *M. spathulata* extracts showed significant (*p* < 0.05) oedema reductions, at all tested doses (100, 200 and 400 mg/kg b.w.) at every time of measurement (30, 60, 120, 180 and 240 min) in comparison with the negative control (vehicle) as is similar to indomethacin at the dosage of 10 mg/kg b.w. This oedema reduction also occurs in a dose-dependent way at 2 and 3 h (*p* < 0.001 with ANOVA) after the induction of inflammation when mice were treated with both extracts. MS_L and MS_B (200 and 400 mg/kg, *p* < 0.05, respectively) showed higher inhibition of paw oedema 2 and 3 h of post-carrageenan induction. Carrageenan is largely utilized as a phlogistic agent to lead to acute inflammation, so the carrageenan-induced paw oedema model is often employed as the method for testing non-steroidal anti-inflammatory (NSAID) agents [58,59]. This protocol is biphasic. The initial phase (1 to 1.5 h) is characterized by non-phagocytic oedema, succeeded by a secondary phase with increased oedema development occurring over the remaining five hours. Different mediators are involved in the various phases of carrageenan-induced oedema. The early stage (up to the first 1.5 h) is dominated by the release of histamine, serotonin, 5-hydroxytryptamine, and PAF (platelet-activating factor). Kinin is released from 1.5 to 2.5 h in the last phase, inflammation will persist to the fifth hour by lipid-derived eicosanoids (prostaglandin, leukotriene, eicosatetraenoic acid 5-hydroperoxide and others) [60,61].

*M. spathulata* extracts were active in both phases of inflammation, but the activities were more pronounced in the late phase. The highest activity was observed at a dose of 400 mg/kg, with inhibition percentages of 94.25% for MS_L and 96.53% for MS_B at 240 min. Indomethacin (10 mg/kg), a cyclooxygenase inhibitor, can mitigate the carrageenan reaction in the second stage of inflammation (71.89%, 240 min), mainly through prostaglandin mediation [62]. The anti-inflammatory effects of extracts, such as indomethacin, were noted especially in the second stage of inflammation. This might imply that the anti-inflammatory effect is caused by the inhibition of prostaglandin release. Nevertheless, the initial anti-inflammatory effects of MS_L (29.58% at 400 mg/kg) and MS_B (31.58% at 400 mg/kg) against carrageenan-induced inflammation, resulting from the release of histamine, serotonin and kinin-like substances in the initial period (1 h) may not be excluded. The anti-inflammatory property of this plant might be due to the content of polyphenols detected in the plant; indeed, this study revealed a positive correlation (R = 0.998; *p* < 0.05) between TPC and anti-inflammatory activity. Moreover, quinic acid, representing 26% and 16% of the total identified compounds in MS_L and MS_B, respectively, and its derivatives have been reported for their anti-inflammatory activities [63]. The mechanism by which quinic acid exerts its anti-inflammatory activity is uncertain, but it appears to be linked to the inhibition of the proinflammatory nuclear factor kappa B (NF-κB) [64,65] contributing to the anti-inflammatory properties of the extracts. 

#### 2.4.2. Acetic Acid-Induced Writhing

The writhing method was used to evaluate the analgesic activity of *M. spathulata*. This method is broadly used in analgesic studies due to its sensitivity and capacity to detect molecules with both sides of the brain affected [66,67]. The activity exerted by MS_L and MS_B were reported in Table 5. MS_L and MS_B showed a strong analgesic effect on acetic acid-induced twisting response during stretching abdominals and in a dose-dependent manner (*p* < 0.001 with ANOVA, respectively). The maximal inhibition was exerted by both extracts at the dose of 400 mg/kg (77.21% and 71.32%, respectively for MS_L and MS_B), analogous to that of paracetamol at 100 mg/kg (83%, *p* > 0.05, respectively) showing the potent analgesic activity of the aerial parts of this species. Acetic acid injection provokes pain by releasing histamine, serotonin, bradykinin, P, and prostaglandins (PGE2a, PGF2a) [68]. These compounds stimulate peripheral nociceptive neurons, increase vascular permeability, and cause pain visceral with the contraction of the abdominal muscles associated with limb elongation and body lengthening [69,70]. The dose-dependent analgesic activity means an increase in the relative content of secondary metabolites in the tested extract, particularly for quinic acid and its derivatives which have been reported for their analgesic activities [59]. The pharmacological mechanism of action could be linked to the synthesis inhibition and liberation of various internal mediators of inflammation and nociceptive, via the increased concentration of the principal compounds found in each extract.

### 2.5. Acute Toxicity

In the acute toxicity tests of MS_L and MS_B, no evidence of toxicity was observed in mice at doses between 250 and 2000 mg/kg b.w., in skin and fur, respiratory system, food consumption, defecation, and other behaviors, according to OECD [71]. During the three days of monitoring, no unusual behavior or mortality was detected in all treated mice with both extracts. Therefore, the LD_50_ values might be higher than the dose of 2000 mg/kg for MS_L and MS_B. In accordance with the OECD 423 guideline method [71], MS_L and MS_B could be considered as non-toxic supporting the traditional use of *M. spathulata*. The non-toxicity of the plants and their extracts has been confirmed in a number of research studies by OECD protocols [55,72,73].

## 3. Materials and Methods

### 3.1. Plant Material

The bark and leaves of *M. spathulata* were collected in the oriental littoral forest (18°24′27″ S, 49°09′20″ E, 76 m above sea level) near the Ambodikininina Fokotany in Ampasimadinika Commune of the Toamasina II District, 12 km from the Toamasina City. The voucher specimen was deposited at the Institut Malgache de Recherches Appliquées (IMRA) after the identification performed by Dr Benja Rakotonirina. After the collection, leaves and bark were separated before being dried in a fresh and aerated environment, out of the sunlight and away from the soil. Pharmacological analyses were conducted at IMRA, while phytochemical analyses were performed at the University of Turin (Department of Agricultural, Forestry and Food Sciences—DISAFA) in Italy.

### 3.2. Extracts Preparation

Plant powders consisting of leaves or barks (100 g) were extracted in 250 mL of a mixture of MeOH/water (95/5; *v*/*v*) for 24 h at ambient temperature with frequent stirring. The extraction procedure was performed three times. Filtrates were then combined and dried at 40 °C in a Rotavapor (Büchi R114, Essen, Germany) at reduced pressure allowing leaf methanol extract (MS_L) and bark methanol extract (MS_B) of *M. spathulata*.

### 3.3. Animals

The tests were conducted on male and female Swiss albino mice aged between 4 and 5 months with an average weight of 25 ± 5 g. Animals at the IMRA animal house were raised under controlled conditions according to Directive 2010/63/EU of the European Parliament and the Council on the Protection of Animals in Scientific Studies. They were kept in a 12 h dark/12 h light cycle at 25 ± 2 °C and 65 ± 5% relative humidity and were fed a standard diet (1420, Livestock Feed Ltd., Port Louis, Mauritius). Prior to the study, the animals fasted overnight. All procedures were approved by the local ethics committee (No. 03/CEA-IMRA/2019).

### 3.4. Analgesic Writhing Test in Mice

The antinociceptive activities of MS_L and MS_B were evaluated following the writhing test method defined by Koster et al. [74]. Eight groups of five fasted mice were used:

- Group I: negative control or vehicle where mice were treated orally with normal saline solution (0.9%).
- Group II: positive control where mice were treated orally with paracetamol at a dose of 100 mg/kg body weight (b.w.).
- Groups III–V: mice were treated orally with MS_L at a dose of 100, 200, and 400 mg/kg b.w., respectively.
- Groups VI–VIII: mice were treated orally with MS_B at different doses (100, 200, and 400 mg/kg b.w., respectively).

A 100 µL solution of 1.5% acetic acid in 0.9% saline was injected intraperitoneally 1 h after sample administration to induce specific writhing. The writhing number in a period of 5–30 min after the injection of acetic acid was considered. The writhing number of the negative control for each extract was used to calculate the inhibition percentage.

### 3.5. Carrageenan-Induced Mice Paw Oedema Assay

The MS_L and MS_B anti-inflammatory properties were performed with slight modifications using the carrageenan-induced mice paw oedema reported by Morris’s method [75]. This method consists of inhibiting carrageenan-induced oedema in the posterior leg of mice using a plethysmometer (Ugo Basile 7140, Varese, Italy). Five mice composed each group of fasted animals. In total, eight groups were considered:

- Group I: negative control or vehicle where mice were treated orally with normal saline solution.
- Group II: mice were treated with a dose of 10 mg/kg per indomethacin (positive control).
- Groups III–V: mice were orally treated with MS_L with a dose of 100, 200, and 400 mg/kg (b.w.), respectively.
- Groups VI–VIII: mice were treated orally with MS_B at the same doses as groups III-V.

A 100 µL solution of 2% carrageenan in 0.9% saline was injected into the right posterior paw of each mouse 1 h after sample application. Paw volume was measured before the injection and then at 30, 60, 120, and 180 min.

### 3.6. Acute Toxicity Assay

MS_L and MS_B oral acute toxicity was assessed following guideline 423 described by the Organization for Economic Co-operation and Development (OECD, 2002) [71]; this method was slightly modified on mice. Fasted animals were divided into nine groups of five:

- Group I: negative control or vehicle where mice were prepared with normal saline solution.
- Group II–V: mice were orally treated with MS_L with a dose of 250, 500, 1000, and 2000 mg/kg (b.w.), respectively.
- Group VI–IX: mice were orally treated with MS_B with a dose of 250, 500, 1000, and 2000 mg/kg (b.w.)¸ respectively.

Each toxic effect was monitored for 6 h on the treated animals. These effects on the animals were then evaluated for the next 3 days. Changes in some body functions (e.g., food and water intake, urination, tremors, temperature, respiration, constipation, eye, and skin color), together with behavior, were studied.

### 3.7. Total Polyphenolic Content (TPC)

The Folin–Ciocalteu reagent was employed to evaluate the TPC in the leaves and barks of *M. spathulata* [76]. Five g of leaf or bark powders were macerated for 24 h in the dark with 75 mL of a mixture of methanol-water (95/5; *v*/*v*) acidified with hydrochloric acid. The mixture was filtered through Whatman filter paper, 185 mm Ø. Ten µL of filtrates were added to 190 µL of extraction solvent, 1000 µL of Folin–Ciocalteu reagent (diluted 1:10), and 800 µL sodium carbonate (7.5%). The last mixture was left in the dark for 30 min, and the absorbance was read using a UV–visible spectrophotometer (1600-PC, VWR International, Radnor, PA, USA) at 760 nm wavelength. Gallic acid standard solution was prepared at 0.02–0.10 mg/mL, according to Donno et al. [77] and the results were reported as gallic acid equivalents (GAE) per 100 g of dry weight (DW).

### 3.8. Anti-Radical DPPH Assay

MS_L and MS_B free radical scavenging capacity was measured by using the spectrophotometric radical DPPH method adapted from Sreejayan and Rao [78]. Shortly, 25 µL of the sample methanol solution with a concentration ranging from 2 to 100 µg/mL was added to 175 µL of DPPH methanol solution (0.25 mmol/L) in a 96-well microplate. The mixtures were incubated at room temperature (21 ± 1 °C) for 30 min. The absorbance was measured at 517 nm with a spectrophotometer Varioskan, (Thermo Fisher Scientific, Waltham, MA, USA). The methanol solution was used as a reference, while the methanol solution of DPPH was employed as a negative control. The antioxidant reference compound used was gallic acid (2.5–40 µg/mL). The results were reported as the mean inhibitory concentration (IC), which was calculated using the equation IC (%) = 100 × (A_0_ − A_1_)/A_0_. A_0_ and A_1_ represent the absorbance of the negative control and the sample, respectively. The IC_50_ was determined using a linear regression equation of the inhibition percentage and sample concentration of the extract or gallic acid, and the ordinate was the percentage of scavenging capacity from three replicates.

### 3.9. Ferric Reducing Antioxidant Power (FRAP) Assay

The antioxidant capacities of the leaves and the barks of *M. spathulata* were evaluated using the FRAP method developed by Benzie and Strain [79]. This procedure is characterized by the ferric reduction (Fe^3+^) to ferrous ions (Fe^2+^) in a solution containing 2,4,6-tripyridyl-S-triazine (TPTZ). Five g of leaf or bark powders were soaked for 24 h in dark with 75 mL of a mixture of methanol–water (95/5; *v*/*v*) acidified with HCL. The mixture was filtered through Whatman filter paper, 185 mm Ø. Ten µL of filtrates were added to 20 µL of extraction solvent, 900 µL of FRAP reagent (0.3 M acetate buffer pH 3.6/TPTZ 10 mM in 40 mM HCl/20 mM ferric chloride; 84/8/8; *v*/*v*/*v*), 90 µL of pure water. Finally, it was placed at 37 °C in a thermostatic bath for 30 min. Blank was prepared by replacing the sample with the extraction solvent. The absorbance was quantified at a wavelength of 595 nm by using a UV–vis spectrophotometer (model 1600—PC, VWR International, Radnor, PA, USA) [80]. FeSO_4_·7H_2_O standard solution was prepared at 100–1000 µmol/mL, and the results were expressed in millimoles of iron ions (Fe^2+^) per kilogram of dried weight.

### 3.10. HPLC Fingerprint Analysis

#### 3.10.1. Sample Preparation

Leaf or bark powders (5 g) were extracted in 75 mL of a mixture of methanol–water (95/5; *v*/*v*) with HCL for 24 h in the dark. The mixture was filtered through Whatman filter paper (185 mm Ø), and the filtrates were recovered and re-filtered with a GHP filter (45 µm) and kept at 4 °C until analysis.

#### 3.10.2. Chromatographic Analysis

The chromatographic analysis of leaf and bark extracts of *M. spathulata* was carried out using an Agilent 1100 HPLC system (Santa Clara, CA, USA) equipped with a G1311A quaternary pump, a manual injection valve and a 20 µL sample loop coupled to an Agilent GI315D diode array UV/Vis detector. Twenty µL of sample were injected, and the chemical components of samples were separated through a C18 column Phenomenex Kinetex, while an NH2 column SphereClone was used only for sugar analysis [80]. A total of five chromatographic methods described in the Supplementary Materials were used to quantify the polyphenols, the organic acids, the vitamins, and the sugars. The detection of bioactive components in samples was based on the comparison of their UV spectra and retention times to the ones of authentic standards using the same experimental conditions. Calibration curves were established by tracing the peak area (y) against the concentration (x) of each standard. The linearity was then measured by the values of the correlation coefficient (R^2^) [81]. The result of any sample is the mean of three repetitions, expressed as mg/100 g of DW, except for organic acids and sugars (*g*/*g* DW).

### 3.11. Statistical Analysis

All samples were set up and analyzed at least in triplicate. The data were expressed as mean values ± standard deviations or standard error mean values. Results were statistically evaluated using the student’s *t*-test and ANOVA test for comparison of means (SPSS 22.0 software), followed by Tukey’s HSD multiple interval test. Values of *p* < 0.05 are considered as statistically significant. Pearson’s coefficient (R) was utilized to investigate the correlations between phytochemicals, TPC, and antioxidant activity. All positive correlations found were then compared and confirmed using the bioinformatics approach outlined by Gfeller et al. [82], which was used to predict potential targets and pharmacokinetics. To identify the putative targets of key compounds, all chemical structures were prepared and converted into SMILES (Simplified Molecular Input Line Entry System) using ACD/ChemSketch (version 12.0). The compound SMILES were then put into the Swiss Target Prediction network (http://www.swisstargetprediction.ch/, accessed on 8 April 2024), and the resulting targets were obtained.

## 4. Conclusions

This study represents the first investigation of the phytochemical and pharmacological properties of *Morella spathulata*, confirming its importance in traditional Malagasy medicine. Extracts of leaves and bark have demonstrated high contents of total phenolic content compared with other Malagasy species, and bioactive compounds, as well as antioxidant, anti-inflammatory, and analgesic properties due to phenols and organic acids, most notably quinic acid, and benzoic and cinnamic acids, such as ellagic and ferulic acids. The synergy of these molecules, expressed in their positive correlation, could positively influence health. Furthermore, the Swiss Target Prediction platform revealed interesting interactions, indicating that ferulic acid targets carbonic anhydrase (CA2), which plays a crucial role in pH regulation and has implications for anti-inflammatory effects. Instead, ellagic acid was found to interact with G protein-coupled receptor 35 (GPR35), suggesting possible involvement in immune regulation and modulation of inflammation. These results provide preliminary information on the molecular strategies driving the therapeutic effects of the plant and highlight its multiple pharmacological properties. By understanding the molecular targets of bioactive compounds, it may be possible to guide targeted therapies and preventive strategies. However, it is crucial to recognize the conservation challenges that this species faces due to illegal logging and habitat loss. Therefore, simultaneous conservation efforts are needed to study effective propagation and domestication techniques, involve local communities in conservation through educational programs, implement selective harvesting measures, establish protected areas that can mitigate over-exploitation and ensure the long-term availability of *M. spathulata*. Indeed, biotechnological solutions, such as in vitro micropropagation, cell suspension, or callus cultures could offer the possibility of producing plant material on a large scale or extracting bioactive compounds avoiding the use or in some cases the abuse of natural resources. These efforts emphasize a dual commitment to medicinal plant exploration and environmental conservation. Considering the scarcity of research on this species, this study may help to reduce the gap in the understanding of its therapeutic potential and provide the basis for future studies.

## Figures and Tables

**Table 1:** Phytochemical fingerprint of the analyzed samples.

Class	Bioactive Marker	MS_L	MS_B	Identified Compounds: Chemical Structures
		Mean value ± SD	Mean value ± SD	
		(mg/100 g DW)	(mg/100 g DW)	
Cinnamic acids	caffeic acid	n.d.	n.q.	
	chlorogenic acid	n.d.	n.d.	
	coumaric acid	n.q.	n.d.	
	ferulic acid	67.16 ± 0.73 ^a^	49.67 ± 2.09 ^b^	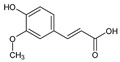
Flavonols	hyperoside	n.q.	n.d.	
	isoquercitrin	n.d.	2.5 ± 0.46	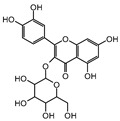
	quercetin	n.d.	n.d.	
	quercitrin	n.d.	n.d.	
	rutin	2.63 ± 1.66	n.d.	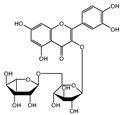
Benzoic acids	ellagic acid	19.08 ± 1.5 ^a^	22.11 ± 2.26 ^a^	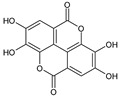
	gallic acid	n.d.	n.d.	
Catechins	catechin	n.d.	n.d.	
	epicatechin	n.d.	n.d.	
Vitamins	ascorbic acid	14.49 ± 0.19	n.d.	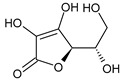
	dehydroascorbic acid	n.d.	n.d.	

MS_L: *M. spathulata* leaf extract; MS_B: *M. spathulata* bark extract. Each value represents the mean ± SD (standard deviation) (N = 3). Significant statistical differences (*p* < 0.05) are highlighted by different letters (a,b). DW = dried weight, n.d. = not detected, n.q. = not quantified.

**Table 2:** Organic acids and sugars of the analyzed samples.

Class.	Bioactive Marker	MS_L	MS_B	Identified Compounds: Chemical Structures
		Mean value ± SD	Mean value ± SD	
		(g/100 g DW)	(g/100 g DW)	
Organic acids	citric acid	n.d.	3.55 ± 0.46	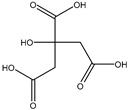
	malic acid	n.d.	n.d.	
	oxalic acid	n.d.	n.d.	
	quinic acid	48.57 ± 0.68 ^a^	21.50 ± 0.75 ^b^	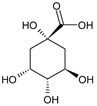
	succinic acid	n.d.	n.d.	
	tartaric acid	n.d.	n.d.	
Sugars	fructose	1.26 ± 0.04 ^b^	1.75 ± 0.03 ^a^	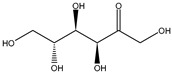
	glucose	24.81 ± 0.76 ^b^	31.44 ± 1.22 ^a^	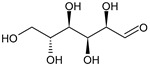
	sucrose	6.04 ± 0.54	n.d.	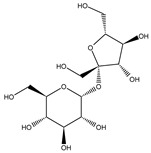

MS_L: *M. spathulata* leaf extract; MS_B: *M. spathulata* bark extract. Each value represents the mean ± SD (standard deviation) (N = 3). Significant statistical differences (*p* < 0.05) are highlighted by different letters (a,b). DW = dried weight, n.d. = not detected.

**Table 3:** FRAP, and IC_50_ values of MS_L and MS_B by DPPH assay.

Sample	FRAP (mmol Fe^2+^/kg DW)	Yield (%)	Equation	r^2^	DPPH Assay: IC_50_ (µg/mL)
MS_L	227.89 ± 12.53 ^a^	3.90	y = 2.0955x − 6.1227	0.9994	26.78 ± 0.42 ^a^
MS_B	376.18 ± 115.69 ^a^	9.84	y = 2.0098x − 9.7684	0.9999	29.74 ± 0.16 ^a^
Gallic acid	-	-	y = 2.7242x − 9.3331	0.9989	21.78 ± 0.11

MS_L: *M. spathulata* leaf extract; MS_B: *M. spathulata* bark extract; DW: dried weight. Each value represents the mean ± SD (standard deviation) (N = 3), ^a^: *p* < 0.05 vs. gallic acid.

**Table 4:** Effects of MS_L and MS_B on carrageenan-induced mouse paw oedema (n = 5).

Sample	Dose (mg/kg)	Inhibition of inflammation (%)				
		30 min	60 min	120 min	180 min	240 min
Vehicle	0	4.28 ± 1.87	9.47 ± 2.52	18.72 ± 2.26	19.99 ± 5.45	32.95 ± 2.77
MS_L	100	11.03 ± 3.21 ^a^	22.59 ± 2.50 ^b^	39.64 ± 3.15 ^b^	62.32 ± 4.44 ^b^	85.74 ± 2.49 ^b,c^
	200	16.85 ± 5.51 ^b^	32.76 ± 6.23 ^b,c^	65.33 ± 6.95 ^b,c^	75.71 ± 7.74 ^b,c^	88.68 ± 5.77 ^b,c^
	400	11.89 ± 1.53 ^a^	29.58 ± 6.84 ^b^	52.44 ± 7.04 ^b^	80.12 ± 6.42 ^b,c^	94.25 ± 1.78 ^b,c^
MS_B	100	6.40 ± 1.15 ^a^	17.43 ± 2.74 ^b^	29.77 ± 2.13 ^b,c^	43.49 ± 3.43 ^b^	49.88 ± 6.64 ^b,c^
	200	12.95 ± 4.05 ^b^	30.21 ± 5.29 ^b^	59.70 ± 3.79 ^b^	74.57 ± 5.19 ^b,c^	91.41 ± 3.57 ^b,c^
	400	12.66 ± 2.83 ^b^	31.58 ± 6.95 ^b^	57.10 ± 8.76 ^b^	85.65 ± 5.83 ^b,c^	96.53 ± 1.90 ^b,c^
Indomethacin	10	13.77 ± 3.89 ^b^	24.02 ± 8.60 ^b^	40.26 ± 7.87 ^b^	57.59 ± 4.14 ^b^	71.89 ± 1.84 ^b^

Values are expressed as mean ± s.e.m of five independent experiments. ^a^: *p* < 0.05 vs. vehicle. ^b^: *p* < 0.01 vs. vehicle. ^c^: *p* < 0.05 vs. indomethacin.

**Table 5:** Effect of MS_L and MS_B on nociceptive responses in the acetic acid-induced writhing test.

Sample	Dose (mg/kg)	Writhing Number	Pain Inhibition (%)
Vehicle	-	13.60 ± 4.46	-
MS_L	100	8.10 ± 2.33 ^a,c^	40.44
	200	4.20 ± 2.93 ^b^	69.12
	400	3.10 ± 0.96 ^b^	77.21
MS_B	100	10.10 ± 1.39 ^c^	25.74
	200	6.00 ± 1.17 ^a,c^	55.88
	400	3.90 ± 0.65 ^b^	71.32
Paracetamol	100	2.60 ± 1.56 ^b^	83.03

Values were expressed as mean ± s.e.m of the writhing number of five independent experiments. ^a^: *p* < 0.05 vs. vehicle. ^b^: *p* < 0.01 vs. vehicle. ^c^: *p* < 0.05 vs. paracetamol.

## Data Availability

The data and results are available to every reader upon reasonable request.

## Supplementary Materials

The following supporting information can be downloaded at: https://www.mdpi.com/article/10.3390/plants13202899/s1, Table S1: Chromatographic conditions of each used method.
