# Supplementary material for: Assessing a “Least-Concern” Red List Tree Species from Madagascar Used in Traditional Medicine: Morella spathulata (Myricaceae) Phyto-Compounds and Anti-Inflammatory Properties
Source: Plants (Basel). 2024 Oct 17;13(20):2899. doi: 10.3390/plants13202899 (PMC11511485; doi:10.3390/plants13202899)
Supplement: Supplementary file 1 [file plants-13-02899-s001.zip › plants-3229385-supplementary.pdf]

**Table S1.** Chromatographic conditions of each used method (Donno et al., 2019).

| Method | Class of Interest | Stationary Phase | Mobile Phase | Wavelength (*nm*) |
|---|---|---|---|---|
| **A** | Cinnamic acid, Flavonols | KINETEX – C18 column (4.6 × 150 mm, 5 μm) | A: 10 mM $KH_2PO_4/H_3PO_4$, pH = 2.8<br>B: $CH_3CN$ | 330 |
| **B** | Benzoic acids, Catechins | KINETEX – C18 column (4.6 × 150 mm, 5 μm) | A: $H_2O/CH_3OH/HCOOH$ (5:95:0.1 v/v/v), pH = 2.5<br>B: $CH_3OH/HCOOH$ (100:0.1 v/v) | 280 |
| **C** | Organic acids | KINETEX – C18 column (4.6 × 150 mm, 5 μm) | A: 10 mM $KH_2PO_4/H_3PO_4$, pH = 2.8<br>B: $CH_3CN$ | 214 |
| **D** | Vitamins | KINETEX – C18 column (4.6 × 150 mm, 5 μm) | A: 5 mM $C_{16}H_{33}N(CH_3)_3Br$/50 mM $KH_2PO_4$, pH = 2.5<br>B: $CH_3OH$ | 261, 348 |
| **E** | Sugars | SphereClone – $NH_2$ column (4.6 × 250 mm, 5 μm) | A: $H_2O$<br>B: $CH_3OH$ | 267, 286 |

*Elutions conditions:*

Method A, gradient analysis: 5%B to 21%B in 17 min + 21%B in 3 min (2 min conditioning time); flow: 1.5 mL $min^{-1}$

Method B, gradient analysis: 3%B to 85%B in 22 min + 85%B in 1 min (2 min conditioning time); flow: 0.6 mL $min^{-1}$

Method C, gradient analysis: 5%B to 14%B in 10 min + 14%B in 3 min (2 min conditioning time); flow: 0.6 mL $min^{-1}$

Method D, isocratic analysis: ratio of phase A and B: 95:5 in 10 min (5 min conditioning time); flow: 0.9 mL $min^{-1}$

Method E, isocratic analysis: ratio of phase A and B: 5:85 in 12 min (3 min conditioning time); flow: 0.5 mL $min^{-1}$
